# Supplementary material for: Content-rich biological network constructed by mining PubMed abstracts
Source: BMC Bioinformatics. 2004 Oct 8;5:147. doi: 10.1186/1471-2105-5-147 (PMC528731; doi:10.1186/1471-2105-5-147)
Supplement: Additional File 2 — The original results of the above study (non-essential files are deleted to keep the file size under the limit set by BMC bioinformatics). [file 1471-2105-5-147-S2.bz2 › chilibotAdditionalFile2/dip40/338ID9228058E4140/html/TNFSF6_TNFRSF6.html]

 


 **TNFSF6** and **TNFRSF6** 
  
Found 4489 abstracts in PubMed, retrieved 40.  
 

 What does Google say? 
 PDF only 
| .edu only 

---

**Interactive relationship** (e.g. stimulation, inhibition, etc)

**Stimulatory relationship**- Furthermore, POH treatment induced a transient G 2 M arrest, enhanced the expression of the membrane bound form of the Fas  [ **TNFRSF6** ]  ligand  [ **TNFSF6** ]  and sensitized the cells to Fas  [ **TNFRSF6** ]  mediated apoptosis.  Ref: 12886519 Prostate, 2003
- Our first paradigmatic inhibitory TSCP, CTLA 4 \* Fas  [ **TNFRSF6** ]  ligand  [ **TNFSF6** ] , binds to APC, and in so doing, simultaneously blocks B7 costimulation via CTLA 4 and sends inhibitory trans signals via Fas  [ **TNFRSF6** ]  ligand  [ **TNFSF6** ]  to T cells with dramatic efficacy.  Ref: 12857999 Immunol Res, 2003
**Neutral relationship**- Apoptosis did not require prior exposure to interleukin 2, nor was it the consequence of CD178  [ **TNFSF6** ]  CD95  [ **TNFRSF6** ]  or TNF TNF R interactions, and was therefore clearly different from activation induced cell death.  Ref: 12893746 BloodBlood, 2003
- T cell maturation in some, but not all, strains of TCR transgenic mice is severely impaired in thymocytes expressing mutant Fas  [ **TNFRSF6** ]  ligand  [ **TNFSF6** ]  incapable of interacting with Fas  [ **TNFRSF6** ] .  Ref: 12860933 J Exp MedJ Exp Med, 2003
- KG1a cells CD34 38 express FAS  [ **TNFRSF6** ]  and TRAIL tumour necrosis factor related apoptosis inducing ligand receptors but are resistant to FAS  [ **TNFRSF6** ]  ligand  [ **TNFSF6** ]  and TRAIL APO2 L apoptosis antigen 2 ligand induced apoptosis.  Ref: 12877678 Br J HaematolBr J Haematol, 2003
- Alterations in expression or shedding of membrane and soluble CD95L  [ **TNFSF6** ]  are associated with numerous diseases, and underscore the pathophysiological relevance of the CD95  [ **TNFRSF6** ]  CD95L  [ **TNFSF6** ]  system.  Ref: 12894217 Cell Death DifferCell Death Differ, 2003

**Non-interactive relationship** (e.g. studied together, co-existance, homology, etc.)

- Role of apoptosis and Fas  [ **TNFRSF6** ]  FasL  [ **TNFSF6** ]  system in the oogenesis of the spotted ray Torpedo marmorata.  Ref: 12874799 Mol Reprod Dev, 2003
- Mutation in Fas  [ **TNFRSF6** ]  Ligand  [ **TNFSF6** ]  Impairs Maturation of Thymocytes Bearing Moderate Affinity T Cell Receptors.  Ref: 12860933 J Exp MedJ Exp Med, 2003
- In conclusion, tolerance induced by TGF beta treated APC appears to be associated with deletion of antigen specific T cells involving the Fas  [ **TNFRSF6** ]  FasL  [ **TNFSF6** ]  pathway.  Ref: 12882832 Int Immunol, 2003
- This study presents a non pharmacological and spatially limited approach for protection of VPC grafted in the CNS against destruction by host immune responses.Murine fibroblast derived VPC expressing herpes simplex virus type I thymidine kinase HSV tk were genetically modified to co express a human Fas  [ **TNFRSF6** ]  ligand  [ **TNFSF6** ]  CD95L  [ **TNFSF6** ]  deletion mutant DeltaFasL resistant to enzymatic cleavage and shedding.  Ref: 12888123 Life Sci, 2003
- Mutant Fas  [ **TNFRSF6** ]  ligand  [ **TNFSF6** ]  inhibits neither negative selection nor death by neglect.  Ref: 12860933 J Exp MedJ Exp Med, 2003
- Fas  [ **TNFRSF6** ]  ligand  [ **TNFSF6** ] , best known as a death inducer, is also a costimulatory molecule required for maximal proliferation of mature antigen specific CD4 and CD8 T cells.  Ref: 12860933 J Exp MedJ Exp Med, 2003
- Soluble Fas  [ **TNFRSF6** ]  CD95  [ **TNFRSF6** ]  Apo 1  [ **TNFRSF6** ] , Soluble Fas  [ **TNFRSF6** ]  Ligand  [ **TNFSF6** ] , and Activated Caspase 3 in the Cerebrospinal Fluid of Infants with Posthemorrhagic and Nonhemorrhagic Hydrocephalus.  Ref: 12867600 Pediatr ResPediatr Res, 2003
- The study aim was to investigate whether anticancer agents can modulate similarly TRAIL receptor and CD95  [ **TNFRSF6** ]  membrane expression and TRAIL and CD95L  [ **TNFSF6** ]  sensitivity.Three colon carcinoma cell lines Caco 2, Colo320 and SW948 were treated with 5 fluorouracil 5 FU, cisplatin or interferon gamma.  Ref: 12865931 Br J Cancer, 2003
- Fas  [ **TNFRSF6** ]  ligand  [ **TNFSF6** ]  on tumor cells mediates inactivation of neutrophils.  Ref: 12874204 J Immunol, 2003
- Overexpression of Bcl 2 may inhibit the Fas  [ **TNFRSF6** ]  FasL  [ **TNFSF6** ]  induced apoptosis.  Ref: 12887767 Zhonghua Yi Xue Za Zhi, 2003
- The expressions of Fas  [ **TNFRSF6** ]  and FasL  [ **TNFSF6** ]  were detected immunohistochemically and quantified by fluorescence activated cell sorting FACS.  Ref: 12875697 Chin Med J (Engl), 2003
- In contrast, the expression of Bax, Fas  [ **TNFRSF6** ]  and Fas  [ **TNFRSF6** ]  ligand  [ **TNFSF6** ]  mRNA and proteins promoters of apoptosis in cardiomyocytes was significantly increased in SAD rats.  Ref: 12859427 Clin Exp Pharmacol PhysiolClin Exp Pharmacol Physiol, 2003
- Immediately after isolation, there was no difference in Bcl 2 or bax expression among the four different samples, or in Fas  [ **TNFRSF6** ]  and FasL  [ **TNFSF6** ]  mRNA.  Ref: 12883827 Arch Dermatol ResArch Dermatol Res, 2003
- OBJECTIVE To evaluate the effects of Carvedilol on cardiopulmonary bypass CPB induced myocardiocyte apoptosis and its effects on regulation of Fas  [ **TNFRSF6** ] , FasL  [ **TNFSF6** ]  expression, caspase 3 activity and oxidative stress in the left ventricle LV in this setting.  Ref: 12875697 Chin Med J (Engl), 2003
- The level of Bcl 2 remained largely unchanged, but the Bax, Fas  [ **TNFRSF6** ] , and FasL  [ **TNFSF6** ]  expression showed up regulation.  Ref: 12880947 Biochim Biophys Acta, 2003
- In contrast, higher expression levels of 12 apoptosis related genes caspase,, 2, BAD, BAX, BID, Fas  [ **TNFRSF6** ] , and FasL  [ **TNFSF6** ]  were shown in chorionic villi from RPL patients than those from normal controls.  Ref: 12874795 Mol Reprod Dev, 2003
- Fas  [ **TNFRSF6** ]  ligand  [ **TNFSF6** ]  is therefore an inducer of death, a costimulator of peripheral T cell activation, and an accessory molecule in positive selection.  Ref: 12860933 J Exp MedJ Exp Med, 2003
- and expression of perforins, Fas  [ **TNFRSF6** ]  ligand  [ **TNFSF6** ] , and other functional markers.  Ref: 12891535 Gastroenterology, 2003
- These results suggest that in these diseases, activated T cells and soluble molecules in portal venous blood may promote Fas  [ **TNFRSF6** ]  FasL  [ **TNFSF6** ]  mediated apoptosis of the bile duct cells and hepatocytes, and contribute to the deterioration in liver function as an inevitable result of positive feedback.  Ref: 12870369 J Int Med ResJ Int Med Res, 2003
- Perforin, granzyme B, and Fas  [ **TNFRSF6** ]  ligand  [ **TNFSF6** ]  were only observed before IVIG administration.  Ref: 12877456 Ann Allergy Asthma Immunol, 2003
- Factors like cytokines, prostaglandine, microbes, MHC molecules, hormones, Fas  [ **TNFRSF6** ]  ligand  [ **TNFSF6** ] , heat shock proteins, phenotypes of dendritic cells and APCs, probably can be polarizing courses of eTLM pathway.  Ref: 12888311 Med Hypotheses, 2003
- In conclusion, in patients affected by cGVHD, ECP induced apoptosis of lymphocytes with the involvement of both the Fas  [ **TNFRSF6** ]  FasL  [ **TNFSF6** ]  system and the Bcl 2 protein family.  Ref: 12883827 Arch Dermatol ResArch Dermatol Res, 2003
- Tumour necrosis factor related apoptosis inducing ligand TRAIL and CD95  [ **TNFRSF6** ]  ligand  [ **TNFSF6** ]  CD95L  [ **TNFSF6** ]  are potent inducers of apoptosis in various tumour cell types.  Ref: 12865931 Br J Cancer, 2003
- Theileria parva Transformed T Cells Show Enhanced Resistance to Fas  [ **TNFRSF6** ]  Fas  [ **TNFRSF6** ]  Ligand  [ **TNFSF6** ]  Induced Apoptosis.  Ref: 12874209 J Immunol, 2003
- The CD95  [ **TNFRSF6** ]  ligand  [ **TNFSF6** ]  is involved as a death factor in the regulation of activation induced cell death, establishment of immune privilege and tumor cell survival.  Ref: 12894217 Cell Death DifferCell Death Differ, 2003
- Cell death was prevented and the ability to expand in vitro was restored by treatment with anti Fas  [ **TNFRSF6** ]  ligand  [ **TNFSF6** ]  FasL  [ **TNFSF6** ]  antibody.  Ref: 12882832 Int Immunol, 2003
- To this end, graft manipulation to reduce the presence of Fas  [ **TNFRSF6** ]  Ligand  [ **TNFSF6** ]  FasL  [ **TNFSF6** ]  expressing cells or interleukin IL 10 and tumor growth factor TGF beta production has been proposed.In contrast to autologous transplantation, graft manipulation during allogeneic transplantation is used extensively.  Ref: 12860168 Int Immunopharmacol, 2003
- The expression of Fas  [ **TNFRSF6** ]  FasL  [ **TNFSF6** ]  has no effect on the gastric mucosa apoptosis related to WRS.  Ref: 12887767 Zhonghua Yi Xue Za Zhi, 2003
- Administration of an antigen Ag per oral route leads to apoptosis of Ag specific CD4 T cells and to development of Th2 cells expressing Fas  [ **TNFRSF6** ]  ligand  [ **TNFSF6** ]  FasL  [ **TNFSF6** ]  in the liver.  Ref: 12883484 Hepatology, 2003
- OBJECTIVE To investigate the correlation between the expressions of apoptosis promoting gene Bax, apotosis inhibiting gene Bcl 2, and Fas  [ **TNFRSF6** ]  FasL  [ **TNFSF6** ]  in gastric mucosa in the course of stress ulcer.  Ref: 12887767 Zhonghua Yi Xue Za Zhi, 2003
- In particular, the Fas  [ **TNFRSF6** ]  receptor and other members of the tumor necrosis factor  [ **TNFSF6** ]  receptors, as well as their respective ligands, have emerged as key regulators of T lymphocyte apoptosis.  Ref: 12857983 Immunol Res, 2003
- As RBC supernatants failed to mobilize intracellular free calcium, the chemoattractants should belong to the group of pure chemoattractants, that is, soluble Fas  [ **TNFRSF6** ]  ligand  [ **TNFSF6** ]  sFasL and transforming growth factor beta1 TGF beta1, known to act without increasing calcium levels.  Ref: 12869488 BloodBlood, 2003
- Upon elimination of the parasite from the host cell by treatment with a theilericidal drug, cells become increasingly sensitive to Fas  [ **TNFRSF6** ]  FasL  [ **TNFSF6** ]  induced apoptosis.  Ref: 12874209 J Immunol, 2003
- CONCLUSIONS Carvedilol significantly reduces CPB induced cardiomyocyte apoptosis in dog hearts and the reduction of cardiomyocyte apoptosis is associated with downregulation of Fas  [ **TNFRSF6** ]  and FasL  [ **TNFSF6** ]  expression, inhibition of caspase 3 activity and oxidative stress in LV.  Ref: 12875697 Chin Med J (Engl), 2003
- 2 Fas  [ **TNFRSF6** ]  mRNA and FasL  [ **TNFSF6** ]  mRNA were not expressed in the controls.  Ref: 12887767 Zhonghua Yi Xue Za Zhi, 2003
- ECP treated lymphocytes of chronic graft versus host disease patients undergo apoptosis which involves both the Fas  [ **TNFRSF6** ]  FasL  [ **TNFSF6** ]  system and the Bcl 2 protein family.  Ref: 12883827 Arch Dermatol ResArch Dermatol Res, 2003
- Thus, WT CFTR expression promotes a rapid expression of CD95  [ **TNFRSF6** ]  CD95  [ **TNFRSF6** ]  ligand  [ **TNFSF6** ]  and apoptotic response to P. aeruginosa infection.  Ref: 12878584 Am J Respir Cell Mol Biol, 2003
- The soluble form of the anti apoptotic regulator Fas  [ **TNFRSF6** ]  sFas and the pro apoptotic factors soluble FasL  [ **TNFSF6** ]  sFasL and activated caspase 3 were studied in the cerebrospinal fluid of infants with hydrocephalus.  Ref: 12867600 Pediatr ResPediatr Res, 2003
- Sensitivity to TRAIL or CD95L  [ **TNFSF6** ]  agonistic anti CD95  [ **TNFRSF6** ]  antibody was determined with cytotoxicity and apoptosis assays.  Ref: 12865931 Br J Cancer, 2003
- The expressions of Fas  [ **TNFRSF6** ]  and FasL  [ **TNFSF6** ]  were significantly downregulated by Carvedilol P <.  Ref: 12875697 Chin Med J (Engl), 2003
- We tested the hypothesis that activation of specific apoptotic pathways represents a mechanism for tubular cell death after CAD kidney transplantation.METHODS Serial tissue sections from paraffin embedded needle biopsy specimens obtained at approximately 1 hr of reperfusion after transplantation of 13 CAD and 12 living related donor LRD renal allografts were examined by using the terminal deoxynucleotide transferase mediated dUTP nick end labeling assay to detect apoptosis and by immunohistochemistry for expression of key pro apoptotic molecules Bax, Bak, tumor necrosis factor  [ **TNFSF6** ]  receptor TNFR 1, Fas  [ **TNFRSF6** ] , and cytochrome c.  Ref: 12865785 Transplantation, 2003
- A liver tolerates a portal antigen by generating CD11c cells, which select Fas  [ **TNFRSF6** ]  Ligand  [ **TNFSF6** ]  Th2 cells via apoptosis.  Ref: 12883484 Hepatology, 2003
- The expression of Fas  [ **TNFRSF6** ]  ligand  [ **TNFSF6** ]  FasL  [ **TNFSF6** ]  on tumor cells tumor FasL  [ **TNFSF6** ]  has been implicated in their evasion of immune surveillance.  Ref: 12874204 J Immunol, 2003
- Expression of mutant non cleavable Fas  [ **TNFRSF6** ]  ligand  [ **TNFSF6** ]  on retrovirus packaging cells causes apoptosis of immunocompetent cells and improves prodrug activation gene therapy in a malignant glioma model.  Ref: 12888123 Life Sci, 2003
- While Fas  [ **TNFRSF6** ]  and FasL  [ **TNFSF6** ]  are involved in apoptosis of bystander cells, mitochondrial pathway is required for apoptosis of infected cells.  Ref: 12888173 Rev Med Interne, 2003
- It also induces fas  [ **TNFRSF6** ]  ligand  [ **TNFSF6** ]  expression, activates procaspase 8 and 9 cleavage, reduces mitochondrial membrane potential, and increases cell death as determined by propidium iodide staining.  Ref: 12876280 J Biol ChemJ Biol Chem, 2003
- Possible promotion of Fas  [ **TNFRSF6** ]  FasL  [ **TNFSF6** ]  mediated apoptosis in the bile duct cells and hepatocyte injury.  Ref: 12870369 J Int Med ResJ Int Med Res, 2003
- To elucidate molecular events in the apoptosis, protein expressions of Bcl 2, Bax, Fas  [ **TNFRSF6** ] , and FasL  [ **TNFSF6** ]  were measured by Western blotting using specific antibodies in HL 60 cells.  Ref: 12880947 Biochim Biophys Acta, 2003
- Pseudomonal infection induced expression of CD95  [ **TNFRSF6** ]  and CD95  [ **TNFRSF6** ]  ligand  [ **TNFSF6** ] , a response that was also delayed in cells homozygous for mutant Cftr alleles.  Ref: 12878584 Am J Respir Cell Mol Biol, 2003
- Immunofluorescence staining together with ordinary and confocal microscopy confirmed the observations that procaspase 3 immunoreactivity was clearly identified in the cytoplasm and nucleus while Fas  [ **TNFRSF6** ]  ligand  [ **TNFSF6** ]  staining was localized to the cell surface and PARP immunoactivity to the nucleus, which were used as controls.  Ref: 12883646 Int J Mol Med, 2003
- Similar processing occurs in TNF, LPS, Fas  [ **TNFRSF6** ]  ligand  [ **TNFSF6** ] , and thapsigargin Tg induced apoptosis.  Ref: 12885762 J Cell BiolJ Cell Biol, 2003
- Encouraging attempts have been made with plasmapheresis, indomethacin, low dose cyclophosphamide, anti CTLA 4, anti FAS  [ **TNFRSF6** ]  ligand  [ **TNFSF6** ]  and, perhaps in the future, more judiciously applied chemotherapy.In contrast to the popular notion that thymic involution cannot be reversed in the adult, studies from the author s laboratory indicate that in aged hydrocortisone stressed mice, a natural Type 1 cytokine mixture IRX 2 hastens the reversal of thymic involution and promotes T cell responses to cytokines and mitogens.  Ref: 12860163 Int Immunopharmacol, 2003
- They constitutively express the death receptor Fas  [ **TNFRSF6** ]  and its ligand, FasL  [ **TNFSF6** ]  but do not undergo apoptosis.  Ref: 12874209 J Immunol, 2003
- CD95  [ **TNFRSF6** ]  ligand  [ **TNFSF6** ]  death factor and costimulatory molecule ? .  Ref: 12894217 Cell Death DifferCell Death Differ, 2003
- We now extend the role of Fas  [ **TNFRSF6** ]  ligand  [ **TNFSF6** ]  by showing that it can also influence thymocyte development.  Ref: 12860933 J Exp MedJ Exp Med, 2003
- Although neither perforin nor Fas  [ **TNFRSF6** ]  ligand  [ **TNFSF6** ]  expression was required for GVHD enhancement, donor IFN gamma production was required for optimal GVHD acceleration in the absence of PD 1 ligation.  Ref: 12874215 J ImmunolJ Immunol, 2003
- In situ hybridization was used to detect the mRNA expression of Bcl 2, Fas  [ **TNFRSF6** ] , and FasL  [ **TNFSF6** ] .  Ref: 12887767 Zhonghua Yi Xue Za Zhi, 2003
- Carvedilol attenuates CPB induced apoptosis in dog heart regulationof Fas  [ **TNFRSF6** ]  FasL  [ **TNFSF6** ]  and caspase 3 pathway.  Ref: 12875697 Chin Med J (Engl), 2003
